# Do Gender and BMI Affect the Motor Skills of Five-Year-Old Preschoolers Differently?

**DOI:** 10.3390/children11070829

**Published:** 2024-07-07

**Authors:** Marcos Mecías-Calvo, Fernando Carregal-San Emeterio, Rubén Navarro-Patón

**Affiliations:** 1 Facultad de Formación del Profesorado, Universidade de Santiago de Compostela, 27001 Lugo, Spain; marcos.mecias@usc.es; 2 Centro de Estudios Deportivos Cántabro (CEDEC), 39587 Camaleño, Spain; fer.carregal@gmail.com

**Keywords:** MABC-2, manual dexterity, aiming and catching, balance, total percentile, children

## Abstract

Background/Objective: Weight gain is associated with numerous health complications and constitutes a serious public health problem. Motor competence (MC) can be a protective factor since children’s participation and practice in physical activities can improve their health. The objective of this study was to investigate the impact of gender and BMI on MC [i.e., manual dexterity (MD), aiming and catching (A&C), balance (Bal), and total MC percentile (TP)] depending on gender (boy/girl) and BMI (normal weight, overweight or obesity). Methods: The MABC-2 battery was applied in a sample of 368 preschool children (5.69 ± 0.28 years of age; 54.9% girls). Results: Boys and girls showed statistically significant differences in MC components: boys had higher scores in A&C (*p* = 0.002), while girls excelled in MD (*p* < 0.001), Bal (*p* = 0.035); TP (*p* < 0.001), and BMI [Bal (*p* = 0.009); TP (*p* = 0.050)], with a higher percentile in those children with overweight in both cases. Statistically significant differences were also found in the interactions between gender*BMI [MD (*p* < 0.001) and TP (*p* < 0.001)]. Conclusions: The findings showed that there were notable variations in total percentile, balance, and manual dexterity between boys and girls. In addition, girls outperformed boys in all categories save aiming and catching. However, males who were overweight or obese earned greater percentiles in both balance and the MABC-2 battery’s total percentile.

## 1. Introduction

The PASOS 2019 study [1] and the ALADINO 2019 study [2] confirmed that in Spain we suffer from an epidemic of childhood obesity that is significantly affecting the development of children and adolescents. As of the latest data, the percentage of schoolchildren with overweight or obesity in Spain has reached 40.6% (23.3% overweight and 17.3% obesity), with overweight being more common in girls (24.7% girls; 21.9% boys) and obesity in boys (15.0% girls; 19.4% boys) [3]. A total of 14.2% of the child and adolescent population suffers from this problematic measure according to the body mass index (BMI), and 24.5% have abdominal obesity. The prevalence of childhood obesity has grown in the last two decades, by 1.6% according to BMI and 8.3% according to abdominal obesity [1]. Weight gain is associated with numerous health complications [2], such as cardiovascular problems, diabetes, and non-communicable diseases in children and adolescents [4]. Furthermore, a healthy body mass index is associated with higher levels of psychological well-being in schoolchildren [5] and constitutes a serious problem that over time increases the risks of poorer future health [6]. Motor competence (MC) can be a protective factor since children’s participation and practice of physical activities can improve their health [7]. Previous studies have not clearly delineated when these differences occur or which factors, such as age, quarter of birth, gender, or BMI, contribute to variations in MC. This study aims to fill these gaps by investigating these variables in a sample of preschool children [8].

Studies on preschoolers’ motor development have shown that environmental and sociodemographic factors, such age or gender, might have an impact [9,10,11]. For instance, girls outperform boys in fine motor skills [12], but boys outperform girls in general skills [13,14]. However, other research shows that there are no gender differences [15], and other studies indicate that girls have better performance in balance [15,16] or better performance in MC than boys [10]. These results reflect a lack of consensus or that the results are inconclusive [17]. The lack of consensus and these discrepancies regarding gender may be due to the fact that, in these studies, the relative age of the children was not taken into account, understanding the relative age effect (RAE) as the fact that children born earlier in the calendar year within a cohort obtain better results than those born later [18]. Additionally, previous studies revealed that children born at the beginning of the school year may have advantages in motor skill development compared to their peers born later in the year [10,19,20]. In this study, the quarter of birth was used as a covariate to try to minimize this effect and verify the effect of gender and BMI.

Another consideration is maturity within the same cohort [10,11,19,21], as these age variances might result in variations in maturity and motor practice experience amongst cohort members [22] and, therefore, favour older children. In recent studies, it has been concluded that children born in the first semester obtain better scores in MC compared to those born in the second semester [10], and differences have even been obtained between those born in the first quarter of the same year and those born in the second, third, and fourth quarters [19]. Regarding BMI, it must be taken into account that low levels of MC and physical activity are positively correlated with greater body fat [23,24]. Scientific evidence regarding BMI indicates that it is a predictor of future motor performance [25] in children and adolescents [26]. Furthermore, recent studies [27] highlight that a high BMI in 5-year-old children contributes to a decrease in MC [28], since children with overweight or obesity may have difficulty moving and may not acquire adequate MC due to their excess weight [29,30].

There is a lack of scientific evidence that has examined the differences between MC, obesity indicators, gender, quarter of birth, and the Movement Assessment Battery for Children-2 (MABC-2), which is commonly used in clinical and research settings and is a good tool to describe MC in a wide range of ages and children, including pre-schoolers. In order to conduct this study, the following research questions were posed: (1) Do the skills and overall percentile of the MABC-2 tests, as well as the gender of the 5-year-old pre-schoolers, differ? According to our theory, boys will have an advantage in balance, aim, and catching, while girls will have an advantage in manual dexterity. (2) Are there variations between the BMI of five-year-old pre-schoolers and the abilities and overall percentile of the MABC-2 tests? We predict that the percentile in each MABC-2 test as well as the overall percentile will decline with increasing degrees of overweight or obesity. Therefore, the aim of this study was to explore what happens to MC [i.e., manual dexterity (MD), aiming and catching (A&C), balance (Bal), and total MC percentile (TP)] regarding gender (boy/girl) and BMI (normal weight, overweight, or obesity) in pre-schoolers.

## 2. Materials and Methods

### 2.1. Study Design

The Movement Assessment Battery for Children-2 (MABC-2) motor skills—manual dexterity, aiming and catching, balance, and the battery’s overall percentile—have been identified as dependent variables in this cross-sectional descriptive design study [25]. The following have been established as independent variables: gender (boy or girl), BMI (normal weight, overweight, or obesity), and quarter of birth (quarter 1, quarter 2, quarter 3, and quarter 4), which has been used as a covariate.

### 2.2. Participants

A total of 400 children were invited to participate, using convenience sampling and geographic proximity according to the subjects who had access to five educational centres in the Galician (Spain) public school system.

The following requirements had to be fulfilled for the students to be included: (1) signed by their parents or legal guardians as an informed consent form; (2) not experiencing any medical or mental health issues that would interfere with the MABC-2 tests’ normal development; (3) students who could not achieve a worldwide percentile greater than five were disqualified because their inability to move properly could have affected the results in MC.

In the end, 368 five-year-old children took part; sixteen were removed for not having provided their parents’ or legal guardians’ informed consent, and sixteen more were removed because their mobility and motor skills were below the battery’s fifth percentile.

Based on their birthdate, each participant was categorized into one of four quarters: quarter 1 (q1: born between January and March), quarter 2 (q2: born between April and June), quarter 3 (q3: born between July and September), and quarter 4 (q4: born between October and December). In accordance with their BMI, they were also divided into two groups: boys (normal weight: 13.0–16.6; overweight: 16.7–18.3; obesity: 18.4 or more) and girls (normal weight: 12.7–16.9; overweight: 17.0–18.9; obesity: 19.0 or more).

### 2.3. Tools

Ad hoc questionnaires were used to gather sociodemographic information such as age, gender, and date of birth.

**Figure 1:**
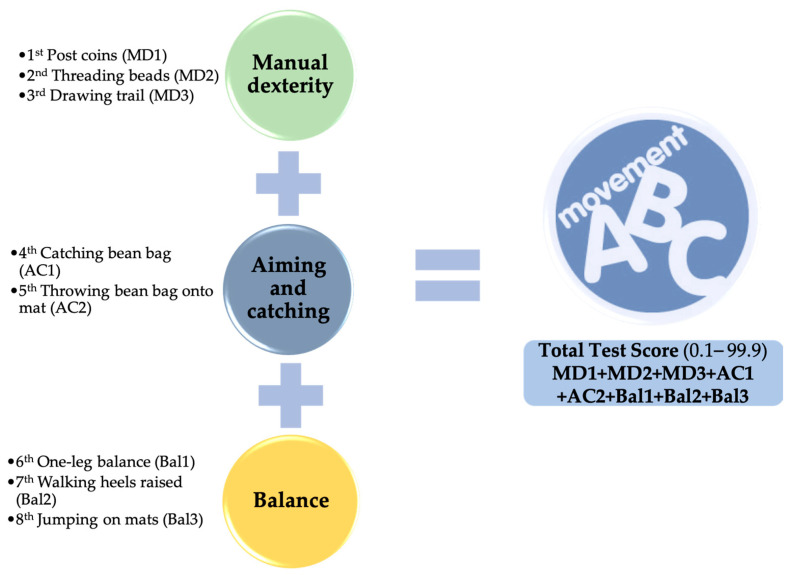
MABC-2 motor skills.

The version of the MABC-2 [9] that Graupera and Ruíz [31] modified for the Spanish context was utilised for motor skills. The MABC-2 assesses motor skills through a series of tasks evaluating manual dexterity, aiming and catching, and balance, providing a comprehensive measure of motor competence. Pre-schoolers can detect changes in their MC with this rigorous and reliable test [9,31,32,33], which has a very good inter-rater reliability [34]. This battery enables the use of eight standardised tests to determine motor competence in three distinct skills (Figure 1). In this sense, the higher the score, the greater the motor competence because a high score in each of the battery’s elements has a positive meaning (less difficulty in executing it) [35]. The percentile score is then calculated for each general motor skill (MD, A&C, and Bal). The battery’s total score (percentile) for each subject was computed using the scores for each motor skill.

### 2.4. Procedures

The initial stage was to convey the study’s goals to the instructors and the administration of the five educational centres. Parents and/or legal guardians were notified and asked to sign an informed consent form for their children’s participation in the research, once the study was approved by the centre and the teaching staff. Next, information from each of the eight MABC-2 battery tests was recorded, together with sociodemographic information such as age and date of birth, gender, weight, and height.

The guidelines of the MABC-2 battery manual were followed, and standardised material was used for data collection. The students were assessed individually by evaluators with previous training in the use of MABC-2 in isolated, bright, clear, and well-ventilated classrooms provided by the educational centres as follows: (1) the task description; (2) the examiner’s demonstration; (3) the child’s practice of the test (during which the examiner could correct any mistakes); and (4) the test’s execution in accordance with the manual’s instructions (no instructions were provided during the test’s performance).

Once the MABC-2 battery was applied, the scale scores of each of the eight tests were calculated, and in this way, the scores (percentiles) of each motor skill and the total percentile score can be calculated.

The national platform EDUCA’s Ethics Committee (code 22019) accepted the research in accordance with the Declaration of Helsinki’s tenets.

### 2.5. Statistical Analysis

The statistical analyses were conducted utilising the IBM Corporation’s SPSS software (version 28, New York, NY, USA). A threshold of statistical significance of *p* < 0.050 was established.

Frequency tables were used to express categorical variables, while measurements of central tendency (mean and standard deviation) were used to convey quantitative variables. The quarter of birth categories (q1 vs. q2 vs. q3 vs. q4) was added as a covariate to eliminate potential confounding factors in order to assess the differences in MABC-2 battery variables regarding gender (boys vs. girls) and BMI (normal weight vs. overweight vs. obesity). The statistical significance was ascertained using the Bonferroni statistic, whereas the effect size was computed using eta squared (η^2^).

## 3. Results

A total of 368 healthy preschool children were evaluated [166 (45.1%) were boys and 202 (54.9%) were girls] with a mean age of 5.69 (SD = 0.28). The distribution of the children by BMI was 124 (33.7%) children with normal weight, 94 (25.5%) children with overweight, and 150 (40.8%) children with obesity. The distribution of the participants by quarter of birth was 66 (17.9%) for q1; 120 (32.6%) for q2; 98 (26.6%) for q3; and 84 (22.8%) for q4. The overall results, by gender and BMI, for each of the specific skills of the MABC-2 battery are shown in Table 1.

### 3.1. Manual Dexterity Results

**Figure 2:**
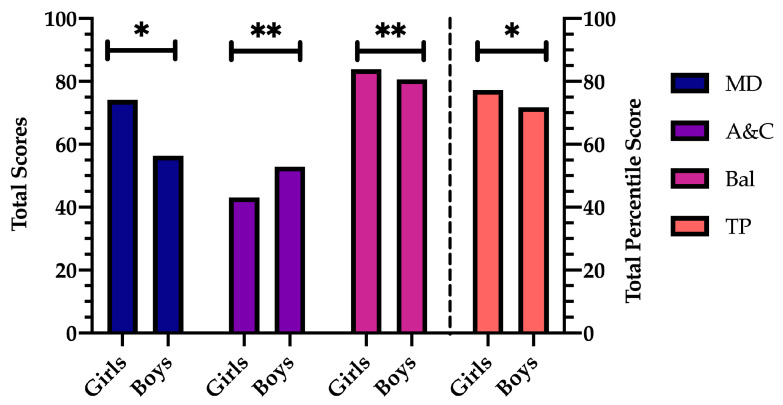
Total percentile skill scores (MD: manual dexterity; A&C: aiming and catching; Bal: balance); and TP: total percentile score according to gender). * *p* < 0.001; ** *p* < 0.05.

**Figure 3:**
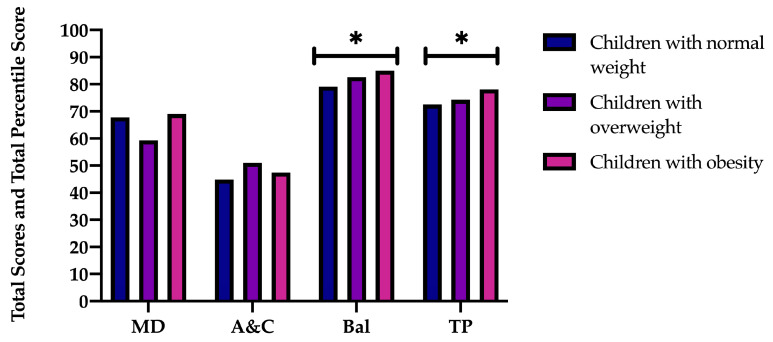
Total percentile skill scores (MD: manual dexterity; A&C: aiming and catching; Bal: balance); and TP: total percentile score according to BMI (children with normal weight vs. children with overweight vs. children with obesity)). * *p* < 0.05.

MANCOVA results for the manual dexterity (MD) test revealed that there is a significant main effect of the gender factor [F (1, 359) = 70.446, *p* < 0.001, η^2^ = 0.164], being lower in boys (Figure 2), but not in the BMI factor (*p* = 0.525) (Figure 3). A significant effect was also found in the interaction of gender and BMI [F (2, 359) = 12.227, *p* < 0.001, η^2^ = 0.064]. These differences occur between boys and girls with normal weight (*p* < 0.001) and overweight (*p* < 0.001), with the percentile of girls being higher. Considering the comparison by gender, significant differences were found between children with overweight and obesity (*p* < 0.001) and those with obesity and normal weight (*p* = 0.050), with boys with obesity having a higher percentile in both cases. Significant differences were found between girls with overweight and girls with obesity (*p* = 0.040), with the former having a higher percentile (Table 1).

### 3.2. Aiming and Catching Results

MANCOVA results in the aiming and catching (A&C) test revealed that there is a significant main effect of the gender factor [F (1, 359) = 9.530, *p* = 0.002, η^2^ = 0.026], being higher in boys (Figure 2), but not in the BMI factor (*p* = 0.375) (Figure 3), nor in the interaction of both factors (*p* = 0.540). However, in pairwise comparisons, the results indicate that boys with overweight have a higher percentile than girls (*p* = 0.014) (Table 1) in this skill.

### 3.3. Balance Results

The results of the MANCOVA in the balance (Bal) test revealed that there is a significant main effect of the gender factor [F (1, 359) = 4.478, *p* = 0.035, η^2^ = 0.012], being lower in boys (Figure 2). A main effect was also found for the BMI factor [F (2, 359) = 4.785, *p* = 0.009, η^2^ = 0.026] (Figure 3), but not in the interaction of gender and BMI (*p* = 0.081). These differences occur between children with normal weight and those with obesity (*p* = 0.008), with a higher percentile in those children with overweight. When gender was taken into account, there were notable differences (*p* = 0.030) between boys and girls with normal weight, with a larger percentile in the case of females. Similar significant differences were observed between children who were normal weight and those who were overweight (*p* = 0.025) and between children who were normal weight and those who were obese (*p* = 0.003), with children who were normal weight having a lower percentile (Table 1).

### 3.4. Total Percentile Results

The results of the MANCOVA regarding the total percentile (TP) revealed that there is a significant main effect of the gender factor [F (1, 359) = 12.965, *p* < 0.001, η^2^ = 0.035], being higher in girls (Figure 2). A main effect was also found for the BMI factor [F (2, 359) = 3.023, *p* = 0.050, η^2^ = 0.017] (Figure 3) and in the interaction of both factors [F (2, 359) = 7.807, *p* < 0.001, η^2^ = 0.042]. When the two groups were compared by gender, the groups of boys and girls who were overweight (*p* = 0.002) and those who were normal weight (*p* < 0.001) differed significantly, with girls in both categories having a higher percentile. Similarly, there were notable distinctions between boys with normal weight and those with obesity (*p* = 0.001), with children who were normal weight having a lower total percentile than obese children (Table 1).

## 4. Discussion

The objective of this study was to investigate the impact of gender and BMI on MC [i.e., manual dexterity (MD), aiming and catching (A&C), balance (Bal), and the total MC percentile (TP)] depending on gender (boy/girl) and BMI (children with normal weight, children with overweight, or children with obesity) in a sample of pre-schoolers. Overall, in response to the study’s first question, “Are there differences in the skills and total percentile of MABC-2 tests and gender of 5-year-old pre-schoolers?”, we have to state that gender does play a role. Boys perform better in object control and manipulation skills compared to girls [10,19,36,37], and girls perform better in fine motor skills [10,12,19,38]. Studies were also found in which girls perform better in balance [10,12,19,38], while others report that girls perform better in motor coordination than boys [39] despite the fact that it is known that boys and girls go through the same sequence of MC development in childhood. These results are inconsistent with those shown in this research [10,19,36,37]. On the other hand, as this study’s results show, girls outperform boys in aiming and catching [6,7,15] and fine motor abilities [8,33], as well as balance [8,33,34]. Depending on the BMI, answering the second question posed in this research [(2) Are there differences in the skills and total percentile of the MABC-2 tests and the BMI of 5-year-old pre-schoolers?], in general, and against what might be expected [40], no differences were found between children with different BMI statuses in MD or A&C, but differences were found in Bal and TP. In addition, schoolchildren with overweight or obesity were those with the highest percentile in these tests, contrary to the results obtained by Musalek et al. [41], who found that children with obesity and overweight had a significantly lower degree of MC than those children with normal weight.

In more detail, the results obtained indicate that girls have a better MC in the two skills studied (i.e., manual dexterity and balance) as well as in the total percentile, which partially coincides with other studies [42]. For example, in terms of manual dexterity, the results coincide with other studies since girls obtain better scores than boys in this motor skill [10,14,30,43]. These results could be explained by the social support that girls experience and by the fact that they are naturally inclined to participate in activities requiring hand-eye coordination and fine motor abilities. According to the study by Webster et al. [44], object manipulation tasks require spatial and temporal synchronization of the limbs rather than complete displacement of body weight, which may explain why the results regarding BMI did not show statistically significant differences with respect to MD. This is important since the interaction between spatial and temporal synchronization in motor tasks is key for efficient motor performance [45], skill learning [46], and different cognitive processes, such as visuospatial ones [47].

Our research’s A&C results indicate a considerable difference between boys and girls [43], with boys achieving greater scores [6,7,15]. This is consistent with findings from earlier research that show boys score higher on manipulation and object control tests [44,45], mostly as a result of the differing sports activities and practice opportunities available to boys and girls. Similar to MD, there are no statistically significant changes seen when analysing the A&C outcomes based on BMI, which is in line with earlier research [48,49]. This could be explained because throwing and object control tasks, like the MD skill, require spatial and temporal synchronisation of limb movements and not a complete displacement of body weight [44].

The girls participating in this study also have a higher percentile than boys in the Bal category. These findings are consistent with other research showing that girls do better in balancing studies [10,12,14]. These findings might be explained by the possibility that girls develop postural control more quickly than boys do [16]. This benefit might only last a short while because this talent doesn’t fully develop until a person is 8 or 9 years old. [50]. Regarding the results obtained in the Bal category in relation to BMI, pre-schoolers with overweight and obesity had better results compared to pre-schoolers with normal weight, which is in contrast to previous studies [48]. This may be the case because children’s ability to move against gravity may be morphologically limited by extra weight, which results in less effort to maintain balance due to the greater effort involved in moving higher body weight [51].

Finally, regarding the TP of the MABC-2 battery, girls once again obtain a better percentile than boys, as occurred in studies such as those by Cheng et al. [39] and Navarro-Patón et al. [10], which reported that girls performed better in MC than boys. Regarding TP and BMI, the significant differences could be explained by the differences in the set of the three tests that make up the MACBC-2 battery. However, differences were identified in the TP of the MABC-2 tests, with a higher percentile in these tests in schoolchildren with overweight or obesity.

We must draw attention to the sample selection, which was not random but rather performed for the convenience of the participating educational institutions, as one of the study’s weaknesses. Furthermore, this corresponds to a single area of Spain, which prevents the results from being generalised. Likewise, the sports practices of pre-schoolers have not been taken into account, which could influence the results obtained; therefore, the results of this research should be taken with caution and not generalised without conducting more research on the matter. Additionally, since there are more accurate methods, we must include as a limitation the use of BMI as a measure of overweight and obesity even though children of all ages with overweight and obesity are frequently measured using BMI.

## 5. Conclusions

The results obtained in this research indicate that girls have better MC in two skills studied (manual dexterity and balance) as well as in the total percentile, while boys have better MC in aiming and catching.

Contrary to expectations, there were no changes in physical dexterity, aiming, or catching skills amongst children with varying BMI states. Overall, the students with overweight or obesity had the highest percentiles in the MABC-2 battery tests.

## Figures and Tables

**Table 1:** MABC-2 results according to BMI and gender.

		Children with Normal Weight		Children with Overweight		Children with Obesity	
		M	SD	M	SD	M	SD
**MD**	boys	59.50	33.31	42.76	31.22	63.97	23.02
	girls	71.76	14.32	79.04	12.65	73.94	28.60
**A&C**	boys	53.20	29.48	57.28	23.07	49.70	26.02
	girls	40.90	28.83	43.71	24.25	45.28	33.38
**Bal**	boys	72.40	18.78	82.12	24.62	84.10	18.60
	girls	82.28	20.30	83.19	22.87	85.94	12.71
**TP**	boys	69.30	29.55	67.92	23.12	78.02	15.12
	girls	74.04	20.34	81.80	11.68	78.28	20.53

Note: M: mean; SD: standard deviation; MD: manual dexterity; A&C: aiming and catching; Bal: balance; TP: total percentile score.

## Data Availability

The data presented in this study are not available in accordance with Regulation (EU) of the European Parliament and of the Council 2016/679 of 27 April 2016 regarding the protection of natural persons with regard to the processing of personal data and the free circulation of these data (RGPD).
